# Supplementary material for: Splice-Junction-Based Mapping of Alternative Isoforms in the Human Proteome
Source: Cell Rep. Author manuscript; Available in PMC 2020 Jan 15. (PMC6961840; doi:10.1016/j.celrep.2019.11.026)
Supplement: 2 [file NIHMS1546469-supplement-2.zip › DF1/PXD010154/Liver/Liver_30_ALB_AAADPHECYAKVLSVVLNQLCVLHEK.pdf]

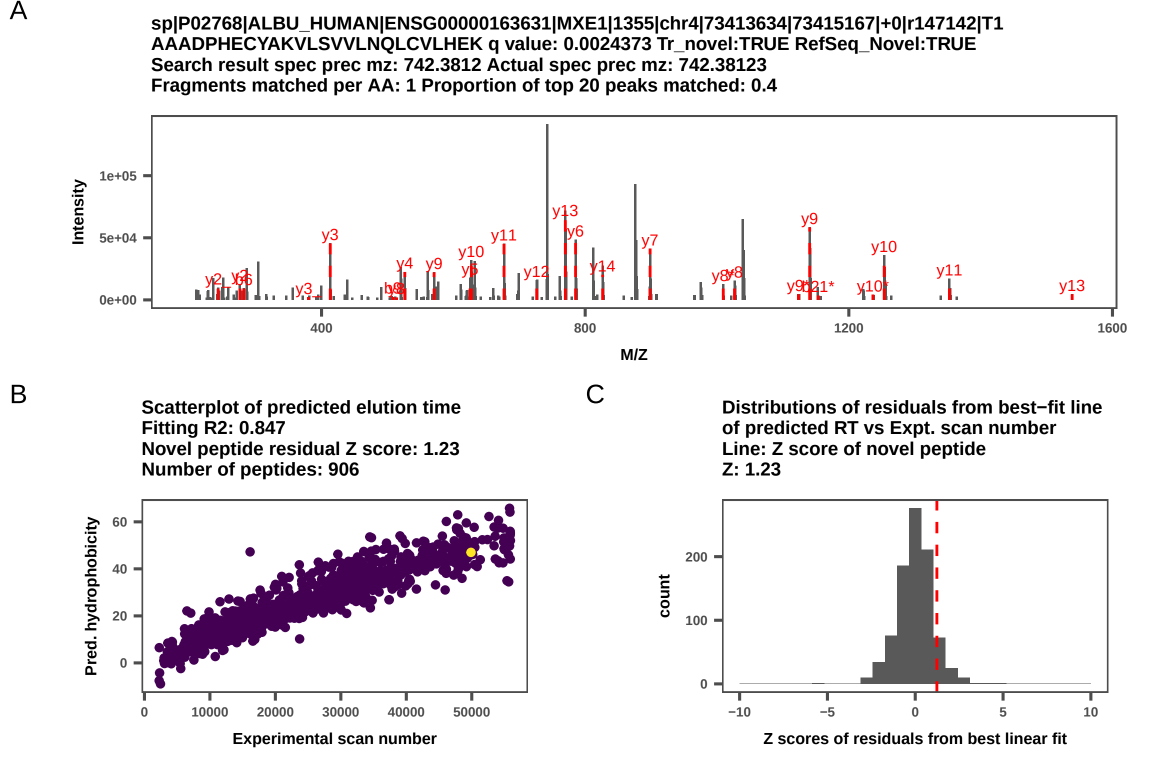
A
sp|P02768|ALBU_HUMAN|ENSG00000163631|MXE1|1355|chr4|73413634|73415167|+0|r147142|T1
AAADPHECYAKVLSVVLNQLCVLHEK q value: 0.0024373 Tr_novel:TRUE RefSeq_Novel:TRUE
Search result spec prec mz: 742.3812 Actual spec prec mz: 742.38123
Fragments matched per AA: 1 Proportion of top 20 peaks matched: 0.4
Intensity
1e+05
5e+04
0e+00
y3
y4
y9
y10
y11
y12
y13
y6
y14
y7
y9
y10
y11
y13
400
800
1200
1600
M/Z
B
Scatterplot of predicted elution time
Fitting R2: 0.847
Novel peptide residual Z score: 1.23
Number of peptides: 906
Pred. hydrophobicity
60
40
20
0
0
10000
20000
30000
40000
50000
Experimental scan number
C
Distributions of residuals from best−fit line
of predicted RT vs Expt. scan number
Line: Z score of novel peptide
Z: 1.23
count
200
100
0
−10
−5
0
5
10
Z scores of residuals from best linear fit
